# Effect of *Angelica sinensis* Root Extract on Cancer Prevention in Different Stages of an AOM/DSS Mouse Model

**DOI:** 10.3390/ijms18081750

**Published:** 2017-08-11

**Authors:** Bochen Zhao, Qian Kang, Yu Peng, Yuanping Xie, Cheng Chen, Bingshao Li, Qing Wu

**Affiliations:** School of Chinese Material Medica, Beijing University of Chinese Medicine, No. 6, Wangjing Zhonghuan Nanlu, Beijing 100102, China; zhaobc2012@163.com (B.Z.); qiankang1989@126.com (Q.K.); pengyupolly@163.com (Y.P.); xieyp_9201@163.com (Y.X.); pangzichencheng@163.com (C.C.); Icyshao@163.com (B.L.)

**Keywords:** *Angelica sinensis*, cancer prevention, antioxidant, P53

## Abstract

*Angelica sinensis* root (ASR) extract was obtained to investigate its effects on colorectal carcinogenesis in different stages of an Azoxymethane/Dextran sodium sulphate (AOM/DSS) model. In this study, we showed that ASR extract administration in the initial stage of the AOM/DSS model had cancer preventive effects with decreasing tumor incidence and a high-grade of intraepithelial neoplasia incidence. With respect to DNA damage, the amounts of 8-oxoguanine and γ-H2AX were suppressed in colon tissue. The balance of apoptosis and proliferation was approaching the normal state. In contrast, ASR extract administration in the promotion stage of the AOM/DSS model accelerated the progression of carcinogenesis. The maximum tumor size reached 49.85 ± 25.04 mm^3^. High-grade pathological changes were significantly increased. Decreased DNA damage and P53 level reflected the disrupted reactive oxygen species (ROS) concentration in colorectal tissue, which led to an imbalance of proliferative and apoptotic relationships. These findings suggested that the cancer-preventive effect of ASR extract may be stage-dependent in the process of carcinogenesis.

## 1. Introduction

Colorectal cancer (CRC) is the third most common cancer globally. However, only about 20% of CRC cases have been associated with heritable genetic changes, and the largest fraction has been linked to environmental factors. On the basis of our present knowledge of risk factors, inflammation bowel disease is closely related to CRC. For this reason, the inhibition of precancerous lesions is a considerable strategy for cancer prevention [1]. The Azoxymethane/Dextran sodium sulphate (AOM/DSS) mouse model is an outstanding platform for colitis-related carcinogenesis and cancer-preventive intervention study. For analogizing human carcinogenesis, the AOM/DSS model is based on a single carcinogenic hit of azoxymethane (AOM) followed by one to three exposition cycles to dextran sodium sulphate (DSS). The model develops through two stages: the initial stage (the acute inflammation stage or the early stage), and the promotion stage [2]. In the early stage (from week 1–7), normal crypts are initiated to form foci of aberrant crypts that proliferate by crypt fission to form micro-adenoma. This initial stage is marked by genetic changes that progress with morphological alternation, involving the Wnt/Apc/β-catenin pathway, K-Ras, c-Myc global hypomethylation, iNOS, and COX-2 [2,3,4,5,6]. Following the resolution of acute inflammation, these micro-adenomas will grow and aggregate to form adenomas, adenomatous polyps, and finally adenocarcinomas in the subsequent weeks (the promotion stage). As described by Tanaka et al. [7], the AOM/DSS model completely reproduced the multi-step process of carcinogenesis characterized by the canonical phases of initiation, promotion, and progression.Different preclinical studies support the use of the AOM/DSS model as aninflammation/oxidative stress-related carcinogenic model to perform the intervention studies of synthetic agents or natural plant compounds. For these reasons, the AOM/DSS model was chosen for our study to evaluate the effect of *Angelica sinensis* root (ASR) extract on cancer prevention.

Traditional Chinese medicine (TCM) employs the dry roots of *Angelica sinensis* (Oliv.) Diels.As a popular herb from umbelliferae, it has been used for medicinal and edible purposes for thousands of years in Asia. Traditionally, *Angelica sinensis*, also known as “female ginseng”, is widely compatible with other herbs for its blood enriching, circulation promoting, and laxative activities [8]. Some scientific reports have indicated the effectiveness of ASR for menopausal symptoms, migraine, anemia, dysmenorrhea, and ulcers [9]. Chemical constituents in ASR show a variety of biological activities including anti-oxidative, anti-inflammatory gastrointestinal protective, and anti-cancer activities [10]. There is also proven evidence of the possible oral and topical safety of ASR for adults [11]. Thus, *Angelica sinensis* has been of interest to researchers recently as a candidate source of agents resistant to oxidative stress-related disease.

Various dietary antioxidant supplements have also shown considerable promise as effective agents for cancer prevention by reducing oxidative stress. Previously, we reported on the cancer preventive potential of ASR extract by protecting cells from oxidative stress in HepG2-C8 and RAW 264.7 cells [12]. However, some contradictory evidence has challenged the above statement. For instance, Sayin et al. [13] reported that the antioxidant agent vitamin E markedly increased tumor progression and reduced survival in the mouse model of B-Raf- and K-Ras-induced lung cancer by disrupting the ROS-P53 axis. As is well-known, evading growth suppressors is one of the hallmarks of carcinogenesis. The circumvention of cellular programed death makes it possible for transformed cells to obtain sustaining proliferation [14]. P53 as an intercellular abnormality sensor that monitors genome damage degree and levels of nucleotide pools, glucose, and oxygenation. If the damage to such cellular systems is irreparable or alarm signals indicate an overwhelming of such systems, P53 can trigger apoptosis. Notably, the activation of P53 is a complex and highly context-dependent progression, which depends on the severity and persistence of the conditions of cell stress and genomic damage [15]. As the occurrence of cancer is a multi-step progression with various contexts of lesion, we suspect that the antioxidants for cancer prevention are stage-dependent. In other words, at specific stages of carcinogenesis, antioxidants protect cells from oxidative stress, and at other stages play an unpredictable role. To verify this assumption, ASR extract was administrated at different stages of carcinogenesis, and its cancer-preventive effect was investigated.

## 2. Results

### 2.1. Angelica Sinensis Root ExtractAdministration in Different Stages of Model Exhibits Opposite Effect on Tumorigenesis

**Figure 1:**
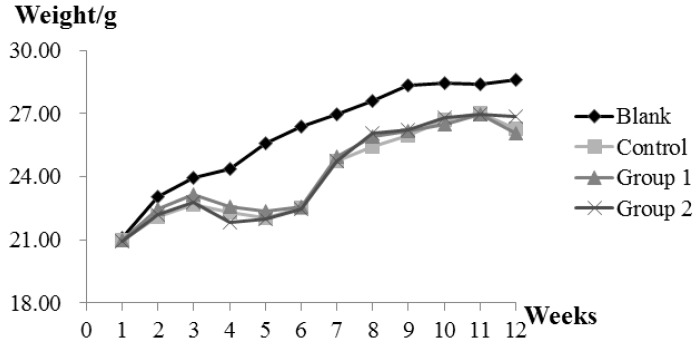
The effect of *Angelica sinensis* root (ASR) extract on body weight in the Azoxymethane/Dextran sodium sulphate (AOM/DSS) mouse model. Body weights across all groups were measured once a week at a fixed time. Experimental treatments of each group are described as Materials and Methods; AOM/DSS Colitis Associated Carcinoma Mouse Model; and Experimental Procedures.

**Figure 2:**
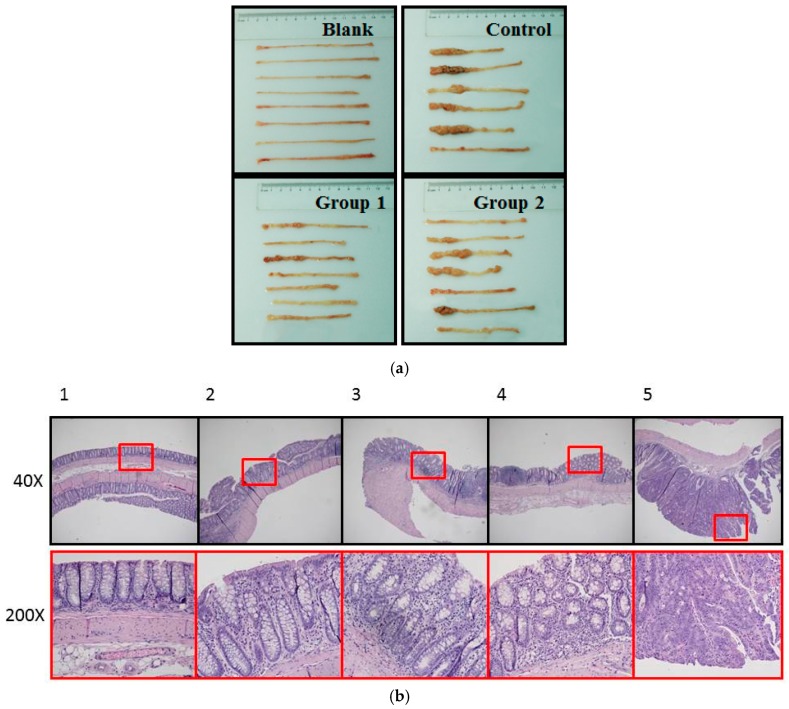
Pathological examination of colorectums of mice in each group. (**a**) Macroscopic examination showed that AOM/DSS treatment led to shorter colorectums. Tumors arose mainly in the distal colon. Colon weight was measured by electronic balance (Sartorius, Göttingen, Germany); (**b**) histopathological examin+ation showed the following five pathological status based on Cooper’s inflammatory grading criteria: 1. Normal gland; 2. Mild inflammation; 3. Moderate inflammation; 4. Low-grade intraepithelial neoplasia (LGIN); 5. High-grade intraepithelial neoplasia (HGIN).

During the experiment, ASR extract administration at a dose of 1 g/kg body weight did not cause any observable toxicity or remarkable weight decline when compared to the control group (Figure 1). Upon macroscopic examination, AOM/DSS treatment led the colon to become shorter and heavier than the blank group. The AOM/DSS model resulted in a 100% incidence of colorectal tumor in the control group. Consistent with the reported studies, the tumors arose mainly in the distal colon (Figure 2a). According to Cooper’s inflammatory grading criteria, mild and moderate inflammations were observed and severe inflammation/ulcerative colitis was absent. Low-grade intraepithelial neoplasia (LGIN) and high-grade intraepithelial neoplasia (HGIN) were discovered in the control groups. Adenocarcinomas were not observed to invade the submucosa, muscularispropria, or serosa (Figure 2b).

**Figure 3:**
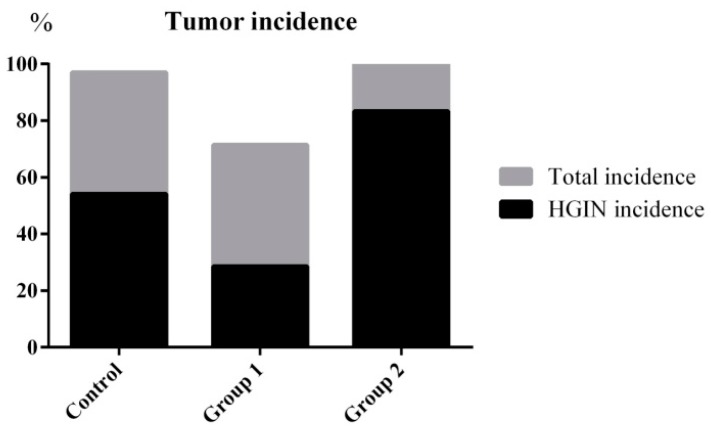
Tumor incidence and HGIN incidence statistical results. ASR extract administration in the initial stage (Group 1) reduced the tumor incidence and HGIN incidence compared with the control group. ASR extract administration in the promotion stage (Group 2) increased the HGIN incidence without inhibiting the tumor incidence.

**Figure 4:**
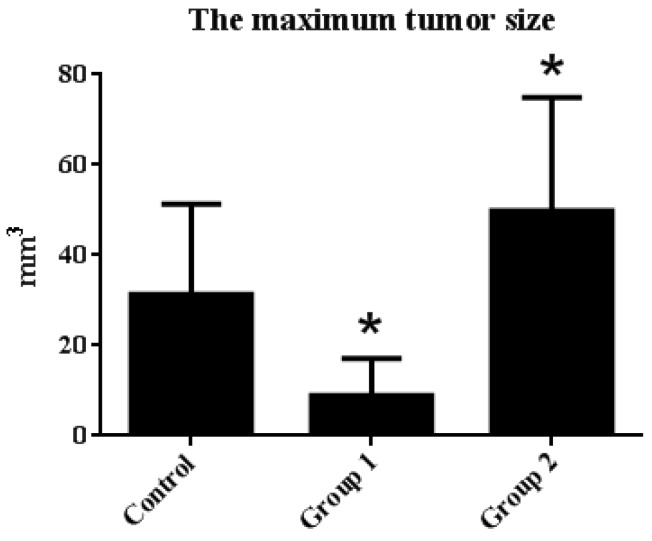
The maximum tumor size statistical results. Tumor diameter was measured by caliper, and the maximum tumor size in each group was calculated by the following equation: tumor size = 3/4 × length × width × height. The results are expressed as means ± S.D.; the amount of sample in each group was *n* = 8 (blank group), *n* = 6 (control group), *n* = 7 (Group 1), *n* = 7 (Group 2). *p* > 0.05 and * *p* < 0.05 when compared with the control group.

The tumor incidence and HGIN incidence of Group 1 declined from 100% and 57.14% to 71.42% and 28.57%, respectively, when compared with the control group. Furthermore, the maximum tumor size was reduced from 31.31 ± 20.01 mm^3^ to 8.92 ± 8.17 mm^3^. On the contrary, without decreasing the tumor incidence, the HGIN incidence increased to 83.33% in Group 2. Although there were no relative changes in colon length, the colon weight of Group 2 was significantly increased when compared with the control group. Correspondingly, the maximum tumor size reached 49.85 ± 25.04 mm^3^ (Figure 3 and Figure 4).

These results suggested that ASR extract administration at different stages of the model exhibited the opposite effect on tumorigenesis. In Group 1, ASR extract administration in the initial stages of carcinogenesis reduced tumor incidence, as well as tumor size. Tumor development was delayed (decreasing HGIN incidence). Notwithstanding, ASR extract treatment in Group 2 promoted tumorigenesis.

### 2.2. The Effect of ASR Extract Administration on Cell Proliferation and Apoptosis

**Figure 5:**
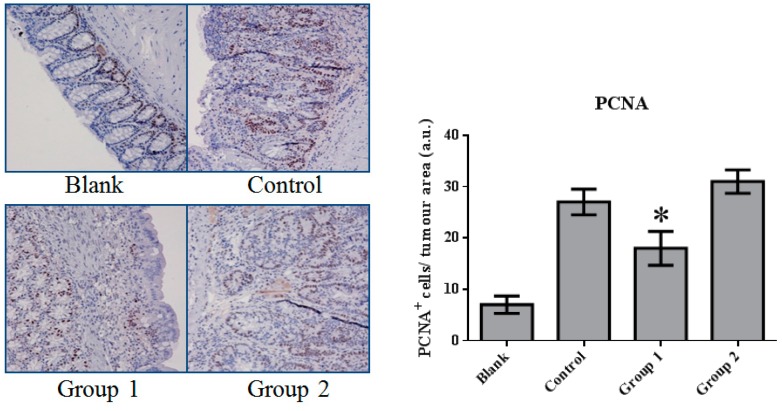
The expression of Proliferating Cell Nuclear Antigen (PCNA) in colorectal tissue. PCNA expression was significantly inhibited by ASR extract treatment in the initial stage (Group 1). The amount of sample in each group was *n* = 8 (blank group), *n* = 6 (control group), *n* = 7 (Group 1), *n* = 7 (Group 2). *p* > 0.05 and * *p* < 0.05 when compared with the control group.

**Figure 6:**
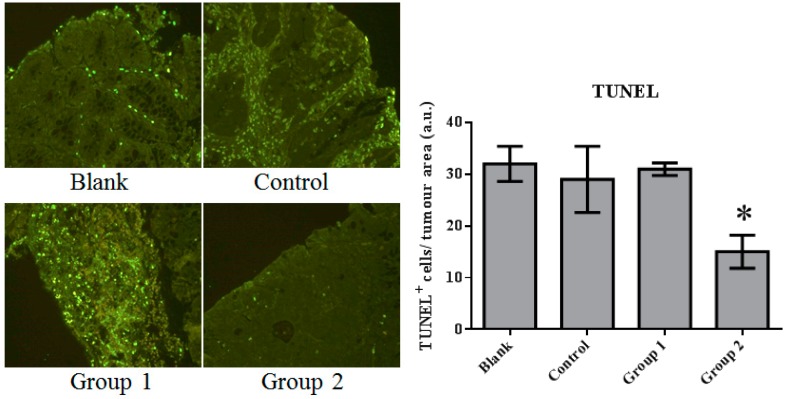
The TUNEL staining of colorectal tissue inspected under fluorescence microscopy. Apoptosis was significantly inhibited by the ASR extract treatment in the promotion stage (Group 2). The amount of sample in each group was *n* = 8 (blank group), *n* = 6 (control group), *n* = 7 (Group 1), *n* = 7 (Group 2). *p* > 0.05 and * *p* < 0.05 when compared with the control group.

The expression of cell proliferation and apoptosis markers were further confirmed in colon sections through immunohistochemistry. Proliferating Cell Nuclear Antigen (PCNA) as an oncologic protein was dramatically upregulated in the control group. Apoptotic signals, evaluated by TUNEL staining, were strengthened in the AOM/DSS-treated mouse colon tissue. Relative to the control group, PCNA expression was depressed and promoted in Groups 1 and 2, respectively (Figure 5). Meanwhile, based on the TUNEL assays, cell apoptosis was essentially suppressed in Group 2 (Figure 6).

These results indicate that AOM/DSS treatment led to the upregulation of cell proliferation and the depression of apoptosis. ASR extract administration at different stages of the carcinogenesis model played completely opposite roles. The macroscopic results were consistent with the conclusion of this part.

### 2.3. ASR Extract Administration Reduces DNA Damage

**Figure 7:**
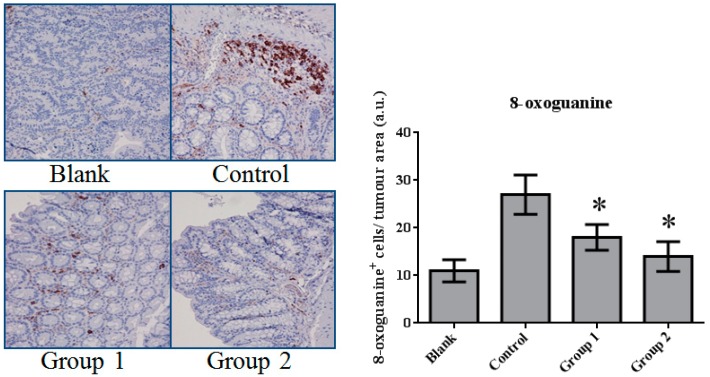
The expression of 8-oxoguanine in colorectal tissue. The 8-oxoguanine expression was significantly inhibited by ASR extract treatment (Groups 1 and 2). The amount of sample in each group was *n* = 8 (blank group), *n* = 6 (control group), *n* = 7 (Group 1), *n* = 7 (Group 2). *p* > 0.05 and * *p* < 0.05 when compared with the control group.

**Figure 8:**
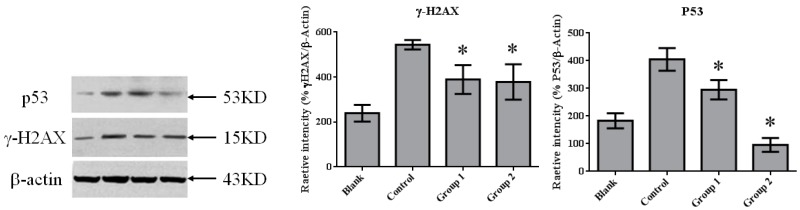
The expression of γ-H2AX and P53 in colorectal tissue. γ-H2AX expression was significantly inhibited by ASR extract treatment (Groups 1 and 2). The amount of sample in each group was *n* = 8 (blank group), *n* = 6 (control group), *n* = 7 (Group 1), *n* = 7 (Group 2). *p* > 0.05 and * *p* < 0.05 when compared with the control group.

DNA damage in colon tissue was determined by 8-oxoguanine and γ-H2AX detection. The positive response of 8-oxoguanine in the colon section was increased in the control group when compared with the blank group (Figure 7); in addition, the level of phosphorylated H2AXser139 (γ-H2AX), determined by Western blot, was also increased (Figure 8). ASR extract administration, whether during the initial stage (Group 1) or the promotion stage (Group 2) of tumorigenesis, reduced the levels of 8-oxoguanine andγ-H2AX markedly. These results showed that ASR extract treatment at different stages of the model produced coordinated effects on DNA protection.

### 2.4. ASR Extract Administration in the Promotion Stage of AOM/DSS Model Downregulated P53 Level

Previous studies have shown that P53 is important in multicellular organisms to prevent cancer formation. Thus, we determined the P53 level in colon tissue by performingWestern blot in each group. In normal colon tissue (the blank group), P53 maintained a relatively lowlevel. AOM/DSS treatment induced colorectal tumor with the dramatic upgrading of P53 in tumor tissue. ASR extract treatment reduced the amount of P53, especially in Group 2 (Figure 8).

## 3. Discussion

This study demonstrated that ASR extract administration either in the initial or promotion stages of the AOM/DSS model reduced amounts of 8-oxoguanine and γ-H2AX in tumors, which indicated that cells were protected from DNA damage. However, these administrations led to opposite effects on carcinogenesis through P53-mediated apoptosis. These results infer that the cancer-preventive effect of ASR extract was stage-dependent, as the antioxidant supplement exerted specific roles at different stages of carcinogenesis.

Recent studies have shown that ASR extracts have an antioxidant potential. Wu et al. [16] reported that *Angelica sinensis* extract inhibited lipid peroxidation in rat liver homogenate in vitro, and exhibited a superoxide anion scavenging activity in a concentration-dependent manner. Cao et al. [17] proved the hepatoprotective and antioxidant effects of *Angelica sinensis* extract (1.0%) against CCl_4_-induced hepatotoxicity in Jian carp. Dietz et al. [18] suggested that *Angelica sinensis* dietary supplements had potential as chemopreventive agents through the induction of Detoxification Enzyme NAD(P)H: Quinone Oxidoreductase 1 by alkylating Keap1 in HepG2-ARE-C8 cells.

Many components from ASRhave also been reported as antioxidant or free radical scavengers. For instance, Kampa et al. [19] reported that ferulic acid and caffeic acid showed a time-dependent and dose-dependent inhibitory effect on T27D human breast cancer cell growth with direct interaction with aryl hydrocarbon receptors and nitric oxide synthase inhibition.Cheng et al. [20] reported that ferulic acid reduces cerebral infarct through its anti-oxidative and anti-inflammatory effects following transient focal cerebral ischemia in rats. Chou et al. [21] reported that vanillic acid inhibited cellular reactive oxygen species in H_2_O_2_-treated BNLCL2 cells.

Previously, our group studied the composition of the ASR extract prepared by our group. FeCl_3_-K_3_(Fe(CN)_6_) UV-VIS spectrophotometric results showed that the phenolic acid content reached 61.4 ± 3.51%, and the HPLC-UV test result showed that ferulic acid content reached 16.14 ± 0.21% (*m*/*m*). Twelve kinds of organic acids were identified using UPLC-MS/MS, including ferulic acid, sassinic acid, chlorogenic acid, camphoric acid, and so on [22].

In this paper, mismatched pairing and DNA double-strand break were reflected by 8-oxoguanine and γ-H2AX levels. These DNA lesions mainly result from reactive oxygen species. We noticed the marked decrease of 8-oxoguanine and γ-H2AX levels after ASR extract administration. These results indirectly show the antioxidative properties of ASR extract.

Oxidative stress mainly results from excessive reactive oxygen species (ROS) [23]. Normally, ROS homeostasis is balanced by the respiratory chain and Nrf2-Keap1 pathway. Pathologically increased levels of ROS can result in the hyperactivation of inflammatory responses and damage to lipids, proteins, and DNA [24]. It is interesting that oxidative stress can also lead to the restriction of the proliferation of damaged cells and the enhancement of DNA repair, or it can induce apoptosis [25].

In our research, cell proliferation activity and apoptosis were characterized by PCNA immunohistochemistry and TUNEL assays. We found that DNA synthesis in Group 1 was inhibited by relatively high apoptotic activity. In contrast, in Group 2, apoptotic activity was significantly suppressed. This variation could be interpreted as the reason for the different effects of ASR extract administration in Groups 1 and 2. Based on the relationship of oxidative stress and apoptosis mediated by P53, Western blot assays were performed. Consistent with the results of the TUNEL assays, P53 expression was significantly suppressed by ASR extract administration that was even lower than the blank group. Studies on P53 revealed that DNA damage was detected by ATM (ataxia telangiectasia mutated) and ATR (ataxia telangiectasia and Rad3 related proteins), which signal downstream to CHK1 and CHK2 (checkpoint kinase) and P53. P53 exerts three crucial functions: regulation and stimulation of DNA repair; signaling cell-cycle checkpoints; and signaling apoptosis. In addition, a certain concentration of ROS acts as both an upstream signal triggering P53 activation and a downstream factor mediating apoptosis [26]. Thus, combined with the component analysis and literature reports, we suggest that ASR extract played an antioxidant role and depleted ROS in epithelial tissue. As a result, ASR extract administration in the promotion stage disrupted the ROS-P53 axis. ASR extract administration in the initial stage reduced the accumulation of DNA damage without the intervention of ROS concentration in the promotion stage, and effectively reduced high-grade pathological changes.

There are studies that have questioned the use of antioxidants, including epidemiological and laboratory studies. Based on our study, we found that antioxidant administration in the initial stage of carcinogenesis could play a positive role, while it promoted carcinogenesis when administrated during the promotion stage. Hence, we hypothesized that effective cancer prevention is based on a clear judgment of precancerous lesions, and antioxidant treatment at an appropriate time is a reasonable choice for inflammation-related carcinogenesis prevention.

In conclusion, it was demonstrated that ASR extract administration in the early stages of the AOM/DSS model prevented DNA damage and reduced tumor incidence, but disrupted P53 activation in later stages, leading to the promotion of carcinogenesis.

In follow-up studies, the composition ofASRextract and the definition of the antioxidant compound, as well as its DNA protectionmechanism, should be further clarified. Furthermore, a clear dose-effect relationship should be studied with a determined ROS clearance timing and degree.

## 4. Materials and Methods

### 4.1. Chemicals and Reagents

Azoxymethane (AOM) was purchased from Sigma-Aldrich (St. Louis, MO, USA) and dextran sodium sulfate (DSS) was purchased from MP Biomedicals (Santa Ana, CA, USA). Antibodies of anti-P53, anti-gamma H2AX (phospho S139), anti-OXoguanine8, and anti-PCNA were purchased from Abcam (Cambridge, UK). Goat anti-mouse IgG (H+L) and goat anti-rabbit IgG (H+L) were purchased from Jackson Immuno-Research (West Grove, PA, USA). The Beta actin antibody was obtained from ZSGB-BIO (Beijing, China). In situ cell death detection kits, POD, were obtained from Roche Ltd. (Laval, QC, Canada). Phosphate buffer saline was obtained from Amresco (Solon, OH, USA). Liquid paraffin and neutral formalin were purchased from Beijing Chemical Works (Beijing, China).

### 4.2. Plant Material and Preparation of ASR Extract

Dry roots of *Angelica sinensis* were purchased from TRT Chinese Materia Medica Company (Beijing, China). Plant identification was undertaken by Professor Liu Chunshen (Faculty of School of Chinese Material Medica, Beijing University of Chinese Medicine, Beijing, China) as per the identification standard of Pharmacopoeia of the People’s Republic of China. The sliced roots of *Angelic sinensis* were extracted with 70% ethanol. The extract was concentrated and centrifuged at 3000× *g* for 30 min to obtain the supernatant. The supernatant was loaded onto an NKA-9 macroporous resin column. To desorb impurities in the extract, the column was eluted by distilled water and 30% ethanol successively. The 45% ethanol eluate was collected and concentrated under vacuum at 60 °C into powder. The content of total organic acids and ferulic acid was determined by the FeCl_3_-K_3_(Fe(CN)_6_) colorimetric method, and the HPLC-UV method reached 61.4 ± 3.51% and 16.14 ± 0.21% (m/m), respectively. The composition of the ASR extract, detected by UPLC-MS/MS, is shown in Table 1, Figure S1, and Table S1.

### 4.3. Azoxymethane/Dextran Sodium Sulphate Colitis-Associated Carcinoma Mouse Model

The animals were maintained in the Beijing University of Traditional Chinese Medicine animal experimental center. Principles of laboratory animal care were followed and all experiments were carried out in accordance with the “Regulation for the Administration of Affairs Concerning Experimental Animals” (State Council of China, 1988). And the Animal Care and Use Committee is the School of Chinese Pharmacy, Beijing University of Chinese Medicine (Date: 7 May 2015; No.: 201505-03). Male 6-week-old Balb/c mice (18–20 g) were purchased from SPF Experimental Animal Technology Co., Ltd. (Beijing, China). Animals were randomly divided into four groups (*n* = 10). Colitis-associated carcinoma was induced by a single-dose intraperitoneally injection of azoxymethane (AOM) (10 mg/kg) in the first week. Dextran sodium sulfate (DSS) (2%) was dissolved in drinking water and administrated in the first, third, and fifth week. Animals were housed under controlled conditions of humidity (50 ± 10%), lighting (12 h light/dark cycle), and temperature (25 ± 2 °C) with pure water and a freely accessible pelleted basal diet. The same procedure was performed with intraperitoneal normal saline and drinking distilled water instead of the AOM/DSS treatment in the blank group.

### 4.4. Experimental Procedures

**Figure 9:**
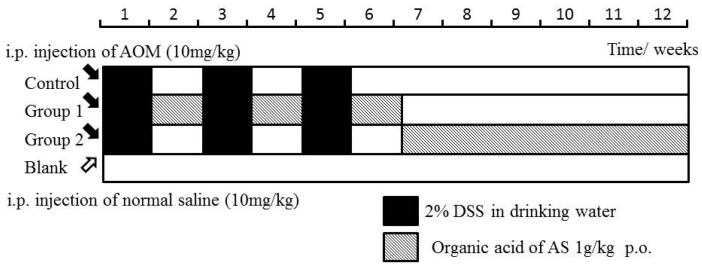
Experimental procedures of the Azoxymethane/Dextran sodium sulphate (AOM/DSS) model establishment and ASR extract administration.

The mice in the control group were not given further treatment. Mice in Groups 1 and 2 were administered by ASR extract orally at different stages of carcinogenesis. The specific method is illustrated in Figure 9. The bodyweight and health condition of the mice were recorded every week. Animals were sacrificed in the 12th week, and large bowels were excised for evaluating histological evidence. All handlings and procedures were carried out as per the protocol approved by the Institutional Animal Care and Usage Committee at Beijing University of Traditional Chinese Medicine.

### 4.5. Hematoxylin-Eosin (HE) Staining and Histological Analysis

The formalin-fixed colon tissues were embedded in paraffin blocks. Sliced sections (4 μm) were deparaffinized and rehydrated by a xylene-ethanol-water gradient system. Hematoxylin and eosin (HE) staining was performed followed by a dehydrating process. Histopathological examination was performed under a light microscope by Olympus (Waltham, MA, USA). Neoplasms and inflammations were analyzed and diagnosed as normal gland, mild inflammation, moderate inflammation, severe inflammation (ulcerative colitis), hyperplasia, aberrant crypt foci (ACF), gastrointestinal intraepithelial neoplasia (GIN), adenoma, and adenocarcinoma as per the established criteria. Histopathological evaluation was determined by two pathologists from the pathology department of Wang-jing Hospital (Chao-yang, Beijing, China) who were not aware of the experimental protocols.

### 4.6. Immunohistochemistry Assay

For immunohistochemical analysis, 4-μm thick sections of colon-rectum tissue were dried and deparaffinized. After antigen retrieving and endogenous peroxidase activity inhibition, the sections were incubated with diluted primary antibodies of 8-oxoguanine (1:10,000) and PCNA (1:5000), and a secondary antibody. Sections were then stained withhaematoxylin slightly for three minutes and dehydrated for observation and photographing under a light microscope.

### 4.7. TUNEL Assay

An in situ cell death detection kit, POD, was applied tothe TUNEL (terminal deoxynucleotidyltransferase-mediated deoxyuridine triphosphate nick end labeling) assay to detect apoptotic cells as per the manufacturer’s instructions. Paraffinized sections were then embedded with the incubation of the TUNEL reaction mixture containingTdT and fluorescein-dUTP. The label incorporated at the damaged sites of the DNA was marked by an anti-fluorescein antibody conjugated with the reporter enzyme POD. Stained sections were examined and photographed under a Nikon Eclipse-Ti Fluorescence microscope system (Tokyo, Japan).

### 4.8. Western Blot Analysis

A 20-mg sample of frozen colon-rectal tissue was homogenized with a 200-μL protease inhibition cocktail and 100 μL lysis buffer for the extraction of total protein. The content of protein was determined by BCA assay, and the protein concentration was adjusted by radio immune-precipitation assay buffer to 5 mg/mL. Equal amounts of protein from tissue lysates were resolved using 10% DSS gel electrophoresis and immunoblotted with primary antibodies (P53 and gamma-H2AX) followed by HRP-conjugated secondary antibodies. After washing the membranes three times for five minutes in TBS buffer containing 0.05% tween-20, the targeted signals were detected on a Li-Cor Odyssey Imager.

### 4.9. Statistical Analysis

Values are presented as means ± SD. Statistical analyses were done by one-way ANOVA test and Fisher’s least significant difference *t*-test. *p* value of less than 0.05 was considered to be statistically significant.

## Figures and Tables

**Table 1:** The chemical composition of the organic acids of *Angelica sinensis* root extract.

No.	Compounds	Molecular Formula
1	Isoeugenol	C_10_H_12_O_2_
2	Sebacic acid	C_10_H_18_O_4_
3	Sassinic acid	C_4_H_6_O_4_
4	Ferulic acid	C_10_H_10_O_4_
5	Anisic acid	C_8_H_8_O_3_
6	Chlorogenic acid	C_16_H_18_O_9_
7	Anchoic acid	C_9_H_16_O_4_
8	Guaiacol	C_7_H_8_O_2_
9	Carvacrol	C_10_H_14_O
10	m-Ethylphenol	C_8_H_10_O
11	Isoeugenol	C_10_H_12_O_2_
12	Camphoric acid	C_10_H_16_O_4_

## Supplementary Materials

Supplementary materials can be found at www.mdpi.com/1422-0067/18/8/1750/s1.
