# Supplementary material for: Effect of Angelica sinensis Root Extract on Cancer Prevention in Different Stages of an AOM/DSS Mouse Model
Source: Int J Mol Sci. 2017 Aug 11;18(8):1750. doi: 10.3390/ijms18081750 (PMC5578140; doi:10.3390/ijms18081750)
Supplement: Supplementary file 1 [file ijms-18-01750-s001.pdf]

Article

Supplementary Material: Effect of *Angelica Sinensis* Root Extract on Cancer Prevention in Different Stages of AOM/DSS Mice Model

Bochen Zhao, Qian Kang, Yu Peng, YuanpingXie, Cheng Chen, Bingshao Li, and Qing Wu

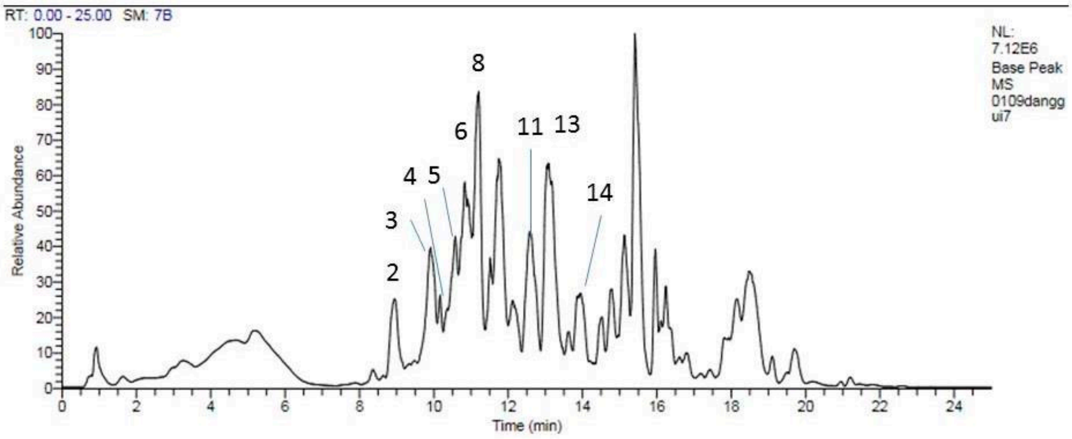

(A)

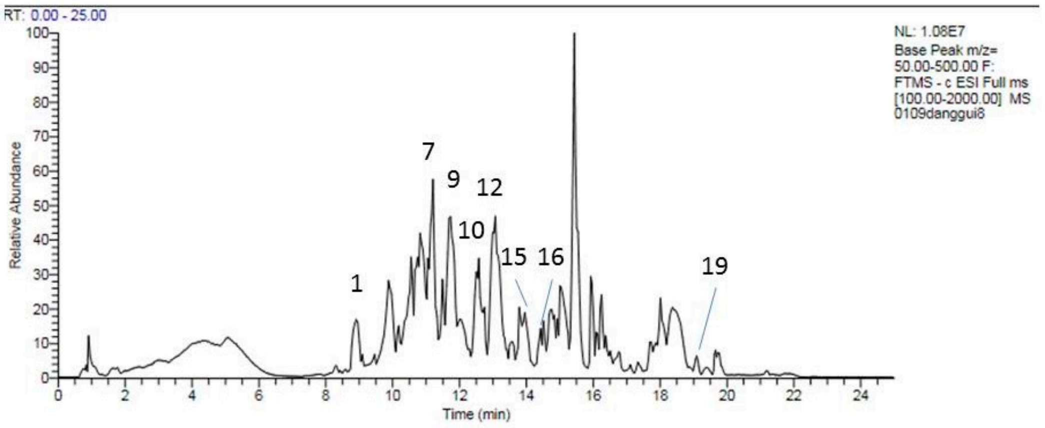

(B)

Figure S1. Total ion-current (TIC) chromatograms ofASR extract within 25 min using UPLC-MS/MS. **A.**The TIC chromatograms in positive APCI mode; **B.** The TIC chromatogramsin negative APCI mode. The peak assignments see Table S1.

Table S1. Characterization of chemical constituents of ASR extract by UPLC-MS/MS.

No.	Precursor ion or adduct ions	Identification	Retention time (min)	Formula	molecular ion peak	Exact mass	Fragment ion
1	[M-H]-	Scopoletin	9.88	$C_{10}H_8O_4$	191.056	192.17	m/z 176; m/z 149
2	[M+H]+	Butylidenephthalide	9.90	$C_{12}H_{14}O_2$	189.092	188.226	m/z 171; m/z 145
3	[M+H]+	Z-Ligustilide	10.05	$C_{12}H_{14}O_2$	191.061	190.242	m/z 173; m/z 147
4	[M+H]+	Isoeugenol	10.59	$C_{10}H_{12}O_2$	165.092	164.204	m/z 147; m/z 135
5	[M+H]+	Sebacic acid	10.84	$C_{10}H_{18}O_4$	203.020	202.25	m/z 184; m/z 165; m/z 147; m/z129
6	[M+H]+	Sassinic acid	11.10	$C_4H_6O_4$	119.051	118.088	m/z 176; m/z 149
7	[M-H]-	Ferulic acid	11.21	$C_{10}H_{10}O_4$	193.050	194.186	m/z 178; m/z 149; m/z 134
8	[M+H]+	Anisic acid	11.39	$C_8H_8O_3$	153.092	152.149	m/z 152; m/z 109
9	[M-H]-	Chlorogenic acid	11.61	$C_{16}H_{18}O_9$	353.088	354.311	m/z 191; m/z 179; m/z 135
10	[M-H]-	Anchoic acid	12.21	$C_9H_{16}O_4$	187.098	188.223	m/z 169; m/z 143; m/z 125
11	[M+H]+	Guaiacol	12.24	$C_7H_8O_2$	125.096	124.139	m/z 107; m/z 123; m/z 81
12	[M-H]-	Vanillin	13.04	$C_8H_8O_3$	150.944	152.149	m/z 151; m/z 136; m/z 123
13	[M+H]+	Scopoletin	13.07	$C_{10}H_8O_4$	193.087	192.17	m/z 176; m/z 149
14	[M+H]+	Carvacrol	14.06	$C_{10}H_{14}O$	151.040	150.221	m/z 133; m/z 109; m/z 107
15	[M-H]-	m-ethylphenol	14.16	$C_8H_{10}O$	120.97	122.167	m/z 123; m/z 105
16	[M-H]-	Isoeugenol	14.43	$C_{10}H_{12}O_2$	163.077	164.204	m/z 149; m/z 135
17	[M-H]-	Camphoric acid	19.85	$C_{10}H_{16}O_4$	199.170	200.234	m/z 183; m/z 155; m/z 109
